# The moderating role of age in the effect of hip and knee joint functions on explosive power performance in soccer players

**DOI:** 10.1186/s13102-026-01865-9

**Published:** 2026-07-15

**Authors:** Halil İbrahim Çakır, Recep Fatih Kayhan, Esranur Terzi, Harun Koç

**Affiliations:** 1 Department of Sports Management, Faculty of Sports Science, Recep Tayyip Erdogan University, Rize, 53020 Türkiye; 2 Department of Coaching Education, Faculty of Sports Science, Marmara University, Istanbul, 34815 Türkiye; 3 Department of Physical Education and Sports, Postgraduate Education Institute, Recep Tayyip Erdogan University, Rize, 53020 Türkiye; 4 Department of Physical Education and Sports Teaching, Faculty of Sports Science, Kutahya Dumlupinar University, Kutahya, 43100 Türkiye

**Keywords:** Age differences, Explosive power, Neuromuscular performance, Soccer players, Youth athletes

## Abstract

**Background:**

Explosive power is a key determinant of soccer performance and is closely associated with lower extremity joint function, particularly hip and knee range of motion (ROM) and muscle strength. These factors contribute to essential actions such as jumping, sprinting, and rapid changes of direction; however, their influence may vary across developmental stages due to age-related neuromuscular and physical differences. Despite existing evidence linking strength and flexibility to motor performance, the moderating role of age in these relationships remains unclear in youth soccer players. Therefore, this study aimed to investigate the effects of hip and knee joint ROM and muscle strength on motor performance and to evaluate the moderating role of age in these associations.

**Methods:**

A total of 59 male soccer players (U15: *n* = 14, U16: *n* = 15, U17: *n* = 15, U19: *n* = 15) from the youth academies of professional teams participated in the study. ROM values of the hip and knee joints, as well as quadricep and hamstring muscle strength, were measured; subsequently, motor performance assessments were conducted. The data were analyzed using multiple regression models including interaction terms with age, and regions of significance were determined using the Johnson–Neyman technique.

**Results:**

For the reactive strength index, hip and knee ROM and quadriceps strength were identified as significant predictors; however, these effects were particularly pronounced in soccer players under the age of 17–18 (r^2^ = 0.076–0.239; *p* < 0.01). Similarly, for vertical and horizontal jump performance, hip and knee ROM, as well as quadriceps and hamstring muscle strength, were significant predictors, with these effects again being more evident in players under the age of 17–18 (r^2^ = 0.061–0.553; *p* < 0.01).

**Conclusion:**

Lower extremity physical characteristics influence motor performance in an age-dependent manner, with stronger effects observed in players under 17–18 years and reduced effects at older ages. These results suggest that training programs for younger age groups may benefit from incorporating range of motion and strength development alongside neuromuscular control exercises, though longitudinal research is needed to confirm causal relationships.

## Introduction

Soccer is a multidimensional sport in which anaerobic power, agility, balance, and a high level of neuromuscular coordination function collectively. By its very nature, the structure of the game involves short-duration, high-intensity efforts such as rapid changes of direction, sudden accelerations, jumping, and leaping. These performance demands cause the physical and neuromuscular characteristics of the lower extremities to play a decisive role in athletic success [1].

Within this framework, muscle strength—one of the key determinants of motor performance—emerges as a parameter subject to developmental changes with age [2, 3]. In particular, the strength levels of the quadriceps and hamstring muscle groups play a critical role in performing high-intensity movements such as running, jumping, and change of direction in soccer players [4]. The strength capacity of these muscles is directly related to the effectiveness of eccentric and concentric contractions, as well as to the functionality of the stretch-shortening cycle [5]. In this context, the Reactive Strength Index (RSI) provides information about the duration and magnitude of power applied to the ground by utilizing the elastic properties of the muscle–tendon complex [6]. In this respect, RSI serves as a neuromechanical output of lower extremity strength and is a valuable indicator in predicting both vertical and horizontal jump performance [6]. While vertical jump assesses explosive power and the capacity to generate power against gravity, horizontal jump is an effective tool for evaluating forward power production and dynamic balance capacity [7]. Accordingly, the inclusion of both vertical and horizontal jump tests in our study enables the assessment of the multidirectional power production capacity of the lower extremities. In addition to muscle strength, joint range of motion (ROM) is another important determinant of motor performance [8]. Adequate ROM allows body segments to operate within optimal movement amplitudes, thereby contributing to the more efficient and controlled execution of motor tasks. Insufficient ROM, on the other hand, may lead to the development of compensatory strategies during movement, resulting in both performance decrements and a potentially elevated injury risk. In particular, adequate range of motion in the hip and knee joints enables the kinetic chain to function effectively and synchronously during tasks such as sprinting, jumping, and changing direction [8].

One of the environmental and biological factors influencing the development of these physical characteristics is age. Neuromuscular changes occurring during adolescence lead to developmental differences in both muscle strength and motor control mechanisms, varying in rate and manner [8]. In this context, age groups (e.g., U15, U16, U17, U19) are likely to play a moderating role in the relationships between physical characteristics and motor performance. Although age-based analyses of this nature have been reported in the literature [9–11], studies that simultaneously address multiple physical characteristics such as ROM and muscle strength and test the effect of age on these relationships using multivariate models are limited [8, 12]. The present study not only compares age groups in terms of motor performance but also provides a multivariate perspective by considering age together with physical variables such as ROM and strength. That is, in our research, age was examined within a framework in which performance was modeled and analyzed alongside other physical determinants. In this regard, the study presents an original structure that evaluates the developmental effects of physical characteristics in conjunction with age (moderating). From this perspective, the study has the potential to make significant contributions to subfields of sport sciences such as developmental physiology, performance analysis, and training planning.

Accordingly, the aim of the present study is to examine, through multivariate models, the effects of lower extremity physical characteristics (muscle strength and joint range of motion) on motor performance indicators (RSI, vertical jump, and horizontal jump) in soccer players from different age groups and to reveal the moderating role of age in these relationships. In line with this objective, it was hypothesized that (i) age significantly affects the relationship between ROM and motor performance, and (ii) age significantly affects the relationship between muscle strength and motor performance.

## Materials and methods

### Study design

This study was structured within a cross-sectional, regression-based moderation framework to elucidate the role of age in shaping the relationship between lower extremity physical attributes (muscle strength and joint ROM) and motor performance outcomes (RSI, vertical and horizontal jump) in youth soccer players. By integrating interaction terms into multivariate models, the design enabled a comprehensive evaluation of age-related differential effects under standardized laboratory conditions.

### Study group

Initially, 72 male academy soccer players were screened for participation. Of these, 13 athletes were excluded for not meeting the predefined inclusion criteria. The final study sample consisted of 59 athletes, allocated into four academy age groups: U15 (*n* = 14), U16 (*n* = 15), U17 (*n* = 15), and U19 (*n* = 15). Although the overall sample size is modest, post-hoc power analysis indicated that 59 participants yielded sufficient statistical power (83.2%) to detect medium-sized interaction effects (f² = 0.15) at the α = 0.05 level. It should be noted, however, that small within-group samples may introduce coefficient instability in interaction models; results should therefore be interpreted with appropriate caution and replicated in larger samples.

Inclusion Criteria:

- Absence of acute or chronic pain in the lower extremities,
- No lower extremity injury within the past 6 months,
- Possession of a valid soccer license,
- At least 3 years of active soccer experience,
- Participation in at least 3 training sessions per week.

### General test procedure

The athletes were informed about the purpose, importance, and content of the tests, instructed to refrain from physical activity beforehand, and asked to wear shorts and a T-shirt. All measurements were conducted in the performance laboratory between 15:00 and 17:00. Prior to testing, height and body mass measurements were obtained, and the athletes’ dominant and non-dominant sides were recorded on the assessment form. Subsequently, the athletes performed a 5-minute warm-up exercise on a cycle ergometer; following this, physical measurements were conducted first, followed by motor performance tests.

#### Muscle strength measurement protocol

Muscle strength measurements were performed using a manual dynamometer (ActivForce 2, ActivForce, USA). Pino-Ortega et al. [13] reported that the ActivForce dynamometer demonstrates high reliability in muscle strength assessments (ICC = 0.913–0.930; Cronbach’s Alpha = 0.920–0.938). Prior to the measurements, in order to familiarize the athletes with the test, a total of four submaximal isometric contractions targeting the quadriceps and hamstring muscle groups were performed on the dominant leg (DL); these trials were not recorded. Subsequently, four maximal isometric contractions were measured for each muscle group. To prevent muscle fatigue, a one-minute rest interval was provided between each trial. For quadriceps muscle strength measurement, the athletes were positioned in a seated posture on an examination table, and the knee extension angle was fixed at 60° using a strap placed at knee level. The dynamometer was positioned on the anterior surface of the lower leg, over the tibia, approximately 5 cm above the lateral malleolus. The athletes were instructed to perform a maximal isometric contraction for at least 5 s by extending their knee [14]. For hamstring muscle strength measurement, the athletes were positioned in a seated posture on the examination table, and the dynamometer was externally stabilized with a safety strap placed 1 cm proximal to the midpoint of the Achilles tendon. The athletes were instructed to perform a maximal isometric contraction for at least 5 s to produce knee flexion [15]. In all measurements, the highest peak isometric muscle strength was recorded in kilograms (kg). Trials in which the athletes were unable to sustain the 5-second contraction were considered invalid and repeated.

#### Range of motion measurement protocol

All measurements were performed using a manual goniometer (Lafayette Instrument Company, USA). The manufacturer reports that this device provides high measurement reliability (ICC = 0.85–0.99) with an accuracy of 1° for angles up to 180° [16]. During ROM measurements, athletes were instructed to remain relaxed and to achieve maximal angles during active movement. Each measurement was repeated three times on the dominant leg, and the average of these measurements was used for analysis. All assessments were conducted by the same researcher, and a standardized protocol was followed to minimize intra-rater variability. For knee flexion ROM measurement, athletes were positioned supine with the knees fully extended. The center of the goniometer was placed over the knee joint; the stationary arm was aligned along the femur, and the movable arm along the tibia. Following alignment, the athlete was instructed to actively flex the knee and draw the heel toward the gluteal region [17]. For hip flexion ROM measurement, athletes were evaluated in the supine position with the knees slightly flexed. The goniometer was positioned at the center of the hip joint; the stationary arm was aligned along the trunk, and the movable arm along the femur. The athlete was then instructed to actively draw the thigh toward the abdomen [17]. For hip extension ROM measurement, athletes were positioned prone. The center of the goniometer was placed over the hip joint; the stationary arm was aligned with the trunk axis, and the movable arm with the femur axis. The athlete was instructed to actively extend the hip by lifting the thigh upward from the ground [17].

#### Reactive strength index and vertical jump height measurement protocol

The drop jump test was administered on the dominant leg using the Jump Mat Pro (FSL Electronics, Northern Ireland). The reported reliability coefficient of the device for drop jump measurement is 0.64 [18]. Athletes stepped off a 30 cm platform onto the electronic mat with one foot and performed a maximal vertical jump with minimal ground contact time, with arms free. Trials were considered invalid and repeated if the knee was excessively flexed upward during the jump or if the landing occurred outside the mat. The test was performed twice with two-minute rest intervals, and RSI (m/s) and jump height data were recorded [18].

#### Standing long jump test protocol

Horizontal jump performance was evaluated using a single-leg horizontal jump test performed with the dominant leg. A line 0.3 m in width was used as the starting point, and a measuring tape 6 m in length and 20 cm in width extending from the midpoint of the line was placed on the ground. During the test, athletes stood on the dominant foot at the marked starting line, jumped forward as far as possible with the same foot, and landed on the same foot. The test was repeated twice for each athlete, and the most successful attempt—measured in centimeters between the starting line and the heel—was recorded. As a criterion for a successful trial, athletes were required to achieve full stabilization upon landing and maintain balance for three seconds [19].

### Data analysis

To determine the required minimum sample size, a power analysis was conducted using G*Power 3.1 (Universität Düsseldorf: Psychologie-HHU). Statistical analyses of the obtained data were performed using IBM SPSS Statistics (IBM).

Prior to analysis, basic assumptions were tested [20]. The normality of residuals was assessed using the Shapiro–Wilk test, and *p* > 0.05 was obtained for all variables. Linearity was examined through scatterplots between dependent and independent variables, and linear relationships were observed. The assumption of homogeneity of variances (homoscedasticity) was evaluated using residuals versus observed values plots, which indicated random distribution of residuals. Autocorrelation was assessed using the Durbin–Watson test, yielding values ranging between 1.83 and 2.10, which were determined to fall within acceptable limits [21]. Multicollinearity was assessed using Variance Inflation Factor (VIF) values; all VIF values fell below the threshold of 5 (range: 1.139–1.721), indicating no problematic collinearity among predictors. Based on these assumptions, it was concluded that Moderated Regression Analysis was applicable.

Accordingly, to test the moderating role of age in the relationship between lower extremity physical characteristics and motor performance, version 4.2 of the PROCESS macro developed by Hayes (Model 1) was employed. In the analyses, bootstrap resampling with 5000 iterations was used to generate estimates within a 95% confidence interval, and interaction terms were evaluated for statistical significance. For significant interactions, simple slope analyses and the Johnson–Neyman procedure were conducted to determine the direction of the interaction and the age levels at which the effect was significant. In all statistical tests, the significance level was set at *p* < 0.05.

Given that the PROCESS macro does not directly provide standardized beta coefficients for interaction models, the relative contribution of predictors was primarily evaluated using unstandardized coefficients (B), standard errors, and 95% confidence intervals reported in Tables 1, 2 and 3. In addition, the incremental explanatory power of interaction effects was assessed using R² change (ΔR^2^) values associated with the inclusion of interaction terms.

To control for potential inflation of Type I error due to multiple moderation models and interaction tests, the Benjamini–Hochberg false discovery rate (FDR) procedure was applied to all interaction effects, with a predefined false discovery rate of q = 0.05. Adjusted p-values were derived based on ranked significance levels to evaluate the robustness of the interaction effects.

## Results

Table 1 shows the multiple regression analyses that examined the effects of age-related interactions between lower extremity ROM and muscle strength variables on RSI, yielding significant models.

**Table 1:** The role of age in the effect of ROM and muscle strength parameters on RSI performance

Predictor (Predicted = RSI)	B	SE	*p*	LLCI	ULCI	*R*^2^/ ΔR^2^	F	*p*
ROM*Age*RSI								
Hip Flexion ROM (cm)	0.064	0.029	0.030*	0.006	0.121	0.196	6.096	0.001**
Age	0.432	0.234	0.069	-0.035	0.899			
Hip Flexion ROM × Age Interaction	-0.003	0.002	0.060	-0.007	0.000	0.039Δ	3.641	0.060
Hip Extension ROM (cm)	0.113	0.038	0.004**	0.037	0.189	0.216	6.870	0.000**
Age	0.235	0.100	0.021*	0.037	0.434			
Hip Extension ROM × Age Interaction	-0.006	0.002	0.008**	-0.010	-0.002	0.076Δ	7.308	0.008*
Knee Flexion ROM (cm)	0.181	0.052	0.001**	0.078	0.234	0.239	7.852	0.000**
Age	1.316	0.412	0.002**	0.496	2.137			
Knee Flexion ROM × Age Interaction	-0.010	0.003	0.002**	-0.016	-0.004	0.107Δ	10.550	0.002**
Strength*Age*RSI								
Quadriceps Strength (kg)	0.077	0.035	0.031*	0.007	0.146	0.207	6.537	0.001**
Age	0.114	0.077	0.144	-0.040	0.269			
Quadriceps Strength × Age Interaction	-0.004	0.002	0.063	-0.008	0.000	0.038Δ	3.567	0.063
Hamstring Strength (kg)	0.106	0.055	0.057	-0.003	0.214	0.201	6.277	0.001**
Age	0.059	0.062	0.348	-0.065	0.183			
Hamstring Strength × Age Interaction	-0.005	0.003	0.115	-0.011	0.001	0.027Δ	2.547	0.115

*CM* Centimeter, *KG* Kilogram, *ROM* Range of motion, *SE* Standard error, *LLCI* Lower limit of the confidence interval, *ULCI* Upper limit of the confidence interval

Significance level (bold) *p < 0.05, **p < 0.01, ΔR^2^ R^2^ Change

Hip flexion ROM demonstrated a significant and positive effect on RSI (B = 0.064, *p* < 0.05). However, the main effect of age (*p* > 0.05) and the hip flexion ROM × age interaction (*p* > 0.05) were not statistically significant. The overall explanatory power of the model was moderate and significant (R² = 0.196; F = 6.096; *p* < 0.01). Although the inclusion of the interaction term with age resulted in a 3.9% increase in explained variance, this contribution was not statistically significant (ΔR^2^ = 0.039; *p* > 0.05). The Johnson–Neyman test indicated that this relationship was significant only in soccer players under 17.82 years of age and lost significance above this age threshold.

Hip extension ROM, age, and the interaction term were all statistically significant (B = 0.113, *p* < 0.01; B = 0.235, *p* < 0.05; B = − 0.006, *p* < 0.01). The overall explanatory power of the model was moderate and significant (R² = 0.216; F = 6.870; *p* < 0.001). The inclusion of the interaction term led to a significant 7.6% increase in explained variance (ΔR^2^ = 0.076; *p* < 0.01). The Johnson–Neyman test revealed that this relationship was significant only in players under 17.76 years of age and became non-significant above this threshold.

Knee flexion ROM, age, and the interaction term were all statistically significant (B = 0.181, *p* < 0.01; B = 1.316, *p* < 0.01; B = − 0.010, *p* < 0.01). The model demonstrated moderate and significant explanatory power (R² = 0.239; F = 7.852; *p* < 0.001). The addition of the interaction term resulted in a significant 10.7% increase in explained variance (ΔR^2^ = 0.107; *p* < 0.01). The Johnson–Neyman analysis indicated that this association was significant only in soccer players under 17.69 years of age.

Quadriceps muscle strength showed a significant positive effect on RSI (B = 0.077, *p* < 0.05). However, the main effect of age (*p* > 0.05) and the quadriceps strength × age interaction (*p* > 0.05) were not statistically significant. The overall model was moderate and significant (R² = 0.207; F = 6.537; *p* < 0.01). Although the interaction term increased the explained variance by 3.8%, this increase was not statistically significant (ΔR^2^ = 0.038; *p* > 0.05). The Johnson–Neyman test indicated that the relationship was significant only in players under 17.62 years of age.

Hamstring muscle strength, age, and their interaction were not statistically significant predictors (*p* > 0.05). Nevertheless, the overall model was moderate and significant (R² = 0.201; F = 6.277; *p* < 0.01). The inclusion of the interaction term led to a 2.7% increase in explained variance, which was not statistically significant (ΔR^2^ = 0.027; *p* > 0.05). The Johnson–Neyman analysis showed that the relationship was significant only in players under 18.23 years of age.

To control for potential inflation of Type I error due to multiple moderation analyses, the Benjamini–Hochberg FDR correction was applied to all interaction effects (q = 0.05). After correction, hip extension ROM × age and knee flexion ROM × age interactions remained statistically significant, whereas hip flexion ROM × age, quadriceps strength × age, and hamstring strength × age did not survive FDR adjustment. These results indicate that age-related moderation effects were more robust in ROM-based models compared to muscle strength-based models.

Table 2 shows that all models were found to be statistically significant as a result of multiple regression analyses regarding the effects of age-related interactions of lower extremity ROM and muscle strength variables on vertical jump performance.

**Table 2:** The role of age in the effect of ROM and muscle strength parameters on vertical jump performance

Predictor (Predicted=Vertical Jump)	B	SE	*p*	LLCI	ULCI	*R*^2^/ ΔR^2^	F	*p*
ROM*Age*Vertical Jump								
Hip Flexion ROM (cm)	2.581	0.713	0.001**	1.161	4.002	0.340	12.872	0.000**
Age	20.533	5.802	0.001**	8.975	32.091			
Hip Flexion ROM × Age Interaction	-0.139	0.042	0.001**	-0.222	-0.055	0.096Δ	10.907	0.001**
Hip Extension ROM (cm)	3.223	0.961	0.001**	1.309	5.136	0.327	12.155	0.000**
Age	8.549	2.519	0.001**	3.532	13.566			
Hip Extension ROM × Age Interaction	-0.170	0.056	0.003**	-0.281	-0.059	0.083Δ	9.279	0.003**
Knee Flexion ROM (cm)	6.513	1.143	0.000**	4.235	8.790	0.500	24.963	0.000**
Age	49.259	9.120	0.000**	31.090	67.427			
Knee Flexion ROM × Age Interaction	-0.349	0.066	0.000**	-0.481	-0.217	0.185Δ	27.729	0.000**
Strength*Age*Vertical Jump								
Quadriceps Strength (kg)	3.291	0.766	0.000**	1.764	4.817	0.488	23.786	0.000**
Age	7.236	1.702	0.000**	3.845	10.626			
Quadriceps Strength × Age Interaction	-0.173	0.046	0.000**	-0.264	-0.082	0.098Δ	14.279	0.000**
Hamstring Strength (kg)	4.792	1.328	0.001**	2.146	7.437	0.366	14.434	**0.000**
Age	5.517	1.517	0.001**	2.495	8.539			
Hamstring Strength × Age Interaction	-0.250	0.078	0.002**	-0.405	-0.096	0.088Δ	10.388	0.002**

*CM* Centimeter, *KG* Kilogram, *ROM* Range of motion, *SE* Standard error, *LLCI* Lower limit of the confidence interval, *ULCI* Upper limit of the confidence interval

Significance level (bold) **p < 0.01, ΔR^2^ R^2^ Change

For hip flexion ROM, age, and the interaction term were all significant (B = 2.581, *p* < 0.01; B = 20.533, *p* < 0.01; B = − 0.139, *p* < 0.01). The model demonstrated high and significant explanatory power (R² = 0.340; F = 12.872; *p* < 0.001). The inclusion of the interaction term resulted in a significant 9.6% increase in explained variance (ΔR^2^ = 0.096; *p* < 0.01). The Johnson–Neyman test indicated significance only in players under 17.60 years of age.

For hip extension ROM, age, and the interaction term were again significant (B = 3.223, *p* < 0.01; B = 8.549, *p* < 0.01; B = − 0.170, *p* < 0.01). The model exhibited high explanatory power (R² = 0.327; F = 12.155; *p* < 0.001), with an 8.3% significant increase in explained variance after adding the interaction term (ΔR^2^ = 0.083; *p* < 0.01). The Johnson–Neyman test indicated significance only in players under 17.85 years of age.

For knee flexion ROM, both main effects and the interaction term were significant (B = 6.513, *p* < 0.001; B = 49.259, *p* < 0.001; B = − 0.349, *p* < 0.001). The model demonstrated high explanatory power (R² = 0.500; F = 24.963; *p* < 0.001), and the interaction term contributed a significant 18.5% increase in explained variance (ΔR^2^ = 0.185; *p* < 0.001). The Johnson–Neyman analysis indicated significance under 18.02 years of age.

Similarly, in the quadriceps strength model, both main effects and the interaction term were significant (B = 3.291, *p* < 0.001; B = 7.236, *p* < 0.001; B = − 0.173, *p* < 0.001). The model showed high explanatory power (R² = 0.488; F = 23.786; *p* < 0.001), with a significant 9.8% increase in explained variance due to the interaction term (ΔR^2^ = 0.098; *p* < 0.001). The Johnson–Neyman test indicated significance only in players under 17.98 years of age.

For hamstring strength, both main effects and the interaction term were also significant (B = 4.792, *p* < 0.01; B = 5.517, *p* < 0.01; B = − 0.250, *p* < 0.01). The model demonstrated high explanatory power (R² = 0.366; F = 14.434; *p* < 0.001), with an 8.8% significant increase in explained variance (ΔR^2^ = 0.088; *p* < 0.01). The Johnson–Neyman test indicated significance only in players under 17.99 years of age.

To control for potential inflation of Type I error due to multiple moderation analyses, a Benjamini–Hochberg FDR correction was applied to all interaction effects (q = 0.05). After correction, all interaction effects in the vertical jump models remained statistically robust, including hip flexion ROM × age (*p* < 0.01), hip extension ROM × age (*p* < 0.01), knee flexion ROM × age (*p* < 0.001), quadriceps strength × age (*p* < 0.001), and hamstring strength × age (*p* < 0.01). These results indicate that all age-related moderation effects on vertical jump performance remained stable after controlling for multiple comparisons, supporting the robustness of both ROM- and strength-based interaction effects.

Table 3 shows the results of multiple regression analyses examining the effects of age-related interactions between lower extremity ROM and muscle strength variables on horizontal jump performance, revealing statistically significant differences in some variables.

**Table 3:** The role of age in the effect of ROM and muscle strength parameters on horizontal jump performance

Predictor (Predicted=Horizontal Jump)	B	SE	*p*	LLCI	ULCI	*R*^2^/ ΔR^2^	F	*p*
ROM*Age*Horizontal Jump Distance								
Hip Flexion ROM (cm)	12.558	6.103	0.043*	0.400	24.716	0.445	20.005	0.000**
Age	66.222	49.658	0.186	-32.701	165.146			
Hip Flexion ROM × Age Interaction	-0.591	0.360	0.104	-1.308	0.125	0.020Δ	2.702	0.104
Hip Extension ROM (cm)	30.991	8.054	0.000**	14.947	47.035	0.457	21.019	0.000**
Age	54.108	21.115	0.012*	12.045	96.171			
Hip Extension ROM × Age Interaction	-1.651	0.468	0.001**	-2.584	-0.718	0.090Δ	12.427	0.001**
Knee Flexion ROM (cm)	37.761	10.892	0.001**	16.064	59.458	0.478	22.918	0.000**
Age	250.909	86.893	0.005**	77.809	424.010			
Knee Flexion ROM × Age Interaction	-1.948	0.632	0.003**	-3.206	-0.689	0.066Δ	9.509	0.003**
Strength*Age*Horizontal Jump Distance								
Quadriceps Strength (kg)	24.852	6.678	0.000**	11.548	38.155	0.553	30.918	0.000**
Age	26.809	14.833	0.075	-2.741	56.358			
Quadriceps Strength × Age Interaction	-1.280	0.399	0.002**	-2.075	-0.484	0.061Δ	10.264	0.002**
Hamstring Strength (kg)	42.144	11.365	0.000**	19.503	64.785	0.466	21.860	0.000**
Age	20.874	12.986	0.112	-4.996	46.743			
Hamstring Strength × Age Interaction	-2.211	0.665	0.001**	-3.535	-0.886	0.079Δ	11.057	0.001**

*CM* Centimeter, *KG* Kilogram, *ROM* Range of motion, *SE* Standard error, *LLCI* Lower limit of the confidence interval, *ULCI* Upper limit of the confidence interval

Significance level (bold) *p < 0.05, **p < 0.01, ΔR^2^ R^2^ Change

Hip flexion ROM showed a significant and positive effect on horizontal jump performance (B = 12.558, *p* < 0.05). However, the main effect of age (*p* > 0.05) and the hip flexion ROM × age interaction (*p* > 0.05) were not significant. The model demonstrated high and significant explanatory power (R² = 0.445; F = 20.005; *p* < 0.001). Although the interaction term led to a 2% increase in explained variance, this contribution was not statistically significant (ΔR^2^ = 0.020; *p* > 0.05). The Johnson–Neyman test indicated significance only below 18.54 years of age.

For hip extension ROM, age and the interaction term were significant (B = 30.991, *p* < 0.001; B = 54.108, *p* < 0.05; B = − 1.651, *p* < 0.01). The model exhibited high explanatory power (R² = 0.457; F = 21.019; *p* < 0.001), with a significant 9% increase in explained variance (ΔR^2^ = 0.090; *p* < 0.01). The Johnson–Neyman analysis indicated significance below 17.84 years of age.

For knee flexion ROM, all main effects and the interaction term were significant (B = 37.761, *p* < 0.01; B = 250.909, *p* < 0.01; B = − 1.948, *p* < 0.01). The model demonstrated high explanatory power (R² = 0.478; F = 22.918; *p* < 0.001), with a significant 6.6% increase in explained variance (ΔR^2^ = 0.066; *p* < 0.01). The Johnson–Neyman analysis indicated significance below 18.23 years of age.

Quadriceps strength significantly and positively affected horizontal jump performance (B = 24.852, *p* < 0.001). The interaction term was also significant (B = − 1.280, *p* < 0.01), whereas the individual effect of age was not (*p* > 0.05). The model showed high explanatory power (R² = 0.553; F = 30.918; *p* < 0.001), with a significant 6.1% increase in explained variance (ΔR^2^ = 0.061; *p* < 0.01). The Johnson–Neyman test indicated significance only in players under 18.14 years of age.

Similarly, hamstring strength was a significant predictor (B = 42.144, *p* < 0.001). Although the interaction term was significant (B = − 2.211, *p* < 0.01), the individual effect of age was not statistically significant (*p* > 0.05). The model demonstrated high explanatory power (R² = 0.466; F = 21.860; *p* < 0.001), with a significant 7.9% increase in explained variance (ΔR^2^ = 0.079; *p* < 0.01). The Johnson–Neyman analysis revealed that the relationship was significant only in players under 17.96 years of age.

To control for potential inflation of Type I error due to multiple moderation analyses, a Benjamini–Hochberg FDR correction was applied to all interaction effects (q = 0.05). After correction, hip extension ROM × age (*p* < 0.01), knee flexion ROM × age (*p* < 0.01), quadriceps strength × age (*p* < 0.01), and hamstring strength × age (*p* < 0.01) remained statistically robust, whereas hip flexion ROM × age (*p* > 0.05) did not survive correction. Overall, four out of five interaction effects remained significant after FDR adjustment, indicating that age-related moderation effects on horizontal jump performance were largely stable, particularly for hip extension ROM, knee flexion ROM, and strength-based variables.

## Discussion

The present study examined the effects of lower extremity ROM and muscle strength on motor performance, with a particular focus on the moderating role of age. Overall, the results suggest that age may have an effect on these relationships, with stronger associations observed in some analyses among younger athletes, although consistent significance was not obtained across all parameters.

In the first result of our study, the effects of ROM and muscle strength variables on RSI were evaluated in interaction with the age factor. In terms of ROM parameters, hip extension and knee flexion ranges of motion demonstrated statistically significant predictive effects on RSI in interaction with age, while hip flexion ROM showed a significant main effect but a non-significant interaction term (*p* > 0.05). The significance of the interaction terms for hip extension and knee flexion ROM indicates that the influence of these ranges of motion on RSI varies depending on age. These results suggest that hip joint mobility, in particular, is associated with more efficient execution during the transition from the amortization phase to the take-off phase, which may be related to the efficiency of the stretch–shortening cycle and RSI performance. According to the Johnson–Neyman analyses, the fact that these effects were significant particularly in athletes under 17–18 years of age suggests that, developmentally, ROM is more determinant for reactive performance at younger ages. The incomplete maturation of neuromuscular coordination during these years may increase the contribution of range of motion to performance [22]. However, with increasing age, the influence of mobility on agility-based outcomes such as RSI appears to diminish, being replaced by strength, speed, and synchronized muscle activation [12].

With regard to strength variables, quadriceps muscle strength significantly and positively predicted RSI, whereas hamstring muscle strength did not demonstrate a significant effect. Although the Johnson–Neyman analysis suggested an association limited to younger athletes, this result does not constitute sufficient evidence for a moderating effect and warrants replication with larger samples before any interpretive conclusions are drawn. This result may be attributed to the quadriceps muscles serving as the primary propulsive power during RSI tasks [23]. In contrast, the supportive role of the hamstring muscles may not be sufficient to independently explain RSI performance [24]. Furthermore, age-related improvements in the direction, timing, and coordination of strength production appear to enhance the contribution of the quadriceps muscles to performance, whereas this effect seems more limited at younger ages [25].

In the second result of our study, the effects of ROM and muscle strength variables on vertical jump performance were evaluated in interaction with age. In terms of ROM parameters, the significance of both the main effects and the age interactions for hip flexion, hip extension, and knee flexion ROM indicates that the contribution of these parameters to vertical jump performance varies according to age. The fact that these effects were significant particularly in individuals under 17–18 years of age suggests that joint range of motion is more determinant for jump performance during developmental stages. In this age group, as synchronized motor unit recruitment, explosive power production, and coordination are not yet fully developed, joint mobility may provide a compensatory advantage [26]. However, the disappearance of these effects with increasing age suggests that, in complex motor outputs such as jumping, ROM is progressively replaced by greater contributions from strength, speed, and neuromuscular control [26].

Regarding strength parameters, the significant interactions of both quadriceps and hamstring muscle strength with age indicate that the influence of muscle strength on vertical jump performance also differs according to age. Since the quadriceps muscle acts as the primary extensor during jumping, increases in strength at younger ages may significantly enhance jump height. However, with advancing age and the development of technical proficiency as well as intra- and inter-muscular coordination, the direct impact of strength on performance appears to weaken [24]. Similarly, the relationship that was significant for hamstring strength at younger ages lost its significance with increasing age. This may be explained by the supportive role of the hamstring muscles during jumping, as their contribution is more related to control, stabilization, and the landing phase [24]. Overall, both ROM and muscle strength variables appear to exert age-sensitive effects on jump performance, with their influence diminishing or being replaced by other motor factors as part of the developmental process.

In the third result of our study, the effects of ROM and muscle strength variables on horizontal jump performance were evaluated in interaction with the age factor. In terms of ROM parameters, hip extension and knee flexion ROM were found to influence horizontal jump performance at different levels depending on age. The fact that these ranges of motion were significant predictors particularly in individuals under 17–18 years of age suggests that joint mobility plays a greater role during the wide propulsion angle and prolonged acceleration phase required in horizontal jumping. Since strength production time is longer in younger age groups, a greater range of motion may support this process and increase jump distance [27]. However, with increasing age, propulsion time shortens and movement efficiency improves, relatively reducing the effect of ROM. Although hip flexion ROM did not demonstrate a significant interaction with age, it was found to be significant in individuals under 18 years of age. This result suggests that hip flexion may provide an advantage for younger athletes during the forward trunk flexion and loading phase required for horizontal jumping [27].

Regarding muscle strength variables, the age-dependent effects observed for both quadriceps and hamstring strength indicate that the role of strength in horizontal jump performance changes throughout the developmental process. Particularly at younger ages, insufficient integration of muscle strength with technical control may cause strength to be reflected more directly in performance [28]. This may explain why quadriceps strength emerged as a significant determinant in athletes under 18 years of age. Similarly, the fact that hamstring strength was significant only in the younger age group may be attributed to the role of these muscles in maintaining the balance between hip extension and knee flexion during jumping, thereby contributing to propulsion efficiency. However, with increasing age and improved movement economy, this contribution becomes less critical [28]. Overall, the effects of ROM and muscle strength variables on horizontal jump performance appear to vary according to age; these physical attributes have a more direct impact at younger ages.

These results are consistent with the literature reporting age-related effects of lower extremity mobility and muscle strength on motor performance. For example, it has been reported that during adolescence, the stretch–shortening cycle improves significantly with the development of the neuromuscular system, with quadriceps muscle strength and lower extremity flexibility playing a determinant role in this improvement [9, 10, 22]. Although both quadriceps and hamstring strength increase with age, muscle imbalances between these groups are frequently observed in younger age groups (particularly 12–16 years), which may contribute to performance limitations and inefficient functioning of the stretch–shortening cycle [23, 25]. Furthermore, the contribution of hip and knee ROM to reactive jump performance provides a more pronounced advantage in age groups where neuromuscular coordination has not yet fully matured [12, 29]. Similarly, vertical jump performance has been shown to be age-sensitive; during the prepubertal period, insufficient elastic energy reutilization limits performance, whereas in adolescence, improvements in muscle–tendon structure and neuromuscular coordination result in marked performance enhancements [11]. In young adulthood, maximal strength production and explosive power capacity further elevate this development to its highest level [11].

On the other hand, it has been reported that during adolescence, hip and knee joint range of motion may exert a more pronounced influence on explosive jump outcomes than strength or neuromuscular proficiency [26]. In particular, in the 12–16 age group, the contribution of such mobility to performance becomes more evident [26]. Additionally, in motor skills such as horizontal jumping, which require a wide propulsion angle and prolonged acceleration, the importance of specific ROM values such as hip extension and knee flexion has been emphasized. It has also been reported that age-related increases in joint mobility positively affect horizontal jump performance in young female athletes [27]. Moreover, it has been suggested that evaluating muscle strength balance and mobility together enables more accurate prediction of jump performance in young athletes [28]. In this context, the age-dependent interaction effects identified in our study are grounded in a biomechanically and developmentally meaningful framework in light of the existing literature.

### Limitations

This study has several limitations. The inclusion of only elite male soccer players affiliated with professional academies limits the generalizability of the results to female athletes, recreational players, and other sports disciplines. Due to its cross-sectional design, the study does not allow for causal inferences. Age groups were classified according to chronological age rather than biological maturation indicators such as peak height velocity (PHV) or bone age. At ages 15–17, inter-individual variation in biological maturity can be substantial, meaning that athletes of the same chronological age may differ markedly in physical development. Future studies should incorporate maturity offset assessments to account for these differences. Additionally, psychological and environmental factors such as sleep, fatigue, and motivation were not controlled. Furthermore, individual training volumes, specific conditioning programs, and injury histories (beyond the six-month exclusion criterion) were not systematically recorded or controlled for, and may represent confounding factors that influenced both physical characteristics and motor performance outcomes. These limitations should be taken into consideration when interpreting the results.

## Conclusion

This study demonstrated that the relationships between lower extremity range of motion, muscle strength, and motor performance (RSI, vertical jump, and horizontal jump) are partially moderated by age in elite male youth soccer players. These associations were more generally pronounced in athletes under 17–18 years of age, suggesting that ROM and strength exert a more direct influence on motor performance outputs during early developmental stages. With advancing age, performance appears to become increasingly dependent on neuromuscular coordination and technical proficiency. These results highlight the importance of age-specific training approaches: in U15–U17 groups, prioritizing multidirectional strength development and ROM maintenance may be particularly beneficial. Longitudinal and experimental studies are needed to establish causal relationships and to generalize results to broader populations.

## Data Availability

The datasets generated and analyzed during the current study are available from the corresponding author upon reasonable request.

## Abbreviations

CM: Centimeter
DL: Dominant leg
FDR: False discovery rate
ICC: Intraclass correlation coefficients
KG: Kilograms
LLCI: Lower limit of the confidence interval
ROM: Range of motion
RSI: Reactive strength index
SE: Standard error
ULCI: Upper limit of the confidence interval
